# Physical activity trajectories and mortality: population based cohort study

**DOI:** 10.1136/bmj.l2323

**Published:** 2019-06-26

**Authors:** Alexander Mok, Kay-Tee Khaw, Robert Luben, Nick Wareham, Soren Brage

**Affiliations:** 1 MRC Epidemiology Unit, University of Cambridge, School of Clinical Medicine, Box 285 Institute of Metabolic Science, Cambridge Biomedical Campus, Cambridge CB2 0QQ, UK; 2 Department of Public Health and Primary Care, University of Cambridge, School of Clinical Medicine, Cambridge Biomedical Campus, Cambridge, UK

## Abstract

**Objective:**

To assess the prospective associations of baseline and long term trajectories of physical activity on mortality from all causes, cardiovascular disease, and cancer.

**Design:**

Population based cohort study.

**Setting:**

Adults from the general population in the UK.

**Participants:**

14 599 men and women (aged 40 to 79) from the European Prospective Investigation into Cancer and Nutrition-Norfolk cohort, assessed at baseline (1993 to 1997) up to 2004 for lifestyle and other risk factors; then followed to 2016 for mortality (median of 12.5 years of follow-up, after the last exposure assessment).

**Main exposure:**

Physical activity energy expenditure (PAEE) derived from questionnaires, calibrated against combined movement and heart rate monitoring.

**Main outcome measures:**

Mortality from all causes, cardiovascular disease, and cancer. Multivariable proportional hazards regression models were adjusted for age, sex, sociodemographics, and changes in medical history, overall diet quality, body mass index, blood pressure, triglycerides, and cholesterol levels.

**Results:**

During 171 277 person years of follow-up, 3148 deaths occurred. Long term increases in PAEE were inversely associated with mortality, independent of baseline PAEE. For each 1 kJ/kg/day per year increase in PAEE (equivalent to a trajectory of being inactive at baseline and gradually, over five years, meeting the World Health Organization minimum physical activity guidelines of 150 minutes/week of moderate-intensity physical activity), hazard ratios were: 0.76 (95% confidence interval 0.71 to 0.82) for all cause mortality, 0.71 (0.62 to 0.82) for cardiovascular disease mortality, and 0.89 (0.79 to 0.99) for cancer mortality, adjusted for baseline PAEE, and established risk factors. Similar results were observed when analyses were stratified by medical history of cardiovascular disease and cancer. Joint analyses with baseline and trajectories of physical activity show that, compared with consistently inactive individuals, those with increasing physical activity trajectories over time experienced lower risks of mortality from all causes, with hazard ratios of 0.76 (0.65 to 0.88), 0.62 (0.53 to 0.72), and 0.58 (0.43 to 0.78) at low, medium, and high baseline physical activity, respectively. At the population level, meeting and maintaining at least the minimum physical activity recommendations would potentially prevent 46% of deaths associated with physical inactivity.

**Conclusions:**

Middle aged and older adults, including those with cardiovascular disease and cancer, can gain substantial longevity benefits by becoming more physically active, irrespective of past physical activity levels and established risk factors. Considerable population health impacts can be attained with consistent engagement in physical activity during mid to late life.

## Introduction

Physical activity is associated with lower risks of all cause mortality, cardiovascular disease, and certain cancers.^1^ ^2^ ^3^ However, much of the epidemiology arises from observational studies assessing physical activity at a single point in time (at baseline), on subsequent mortality and chronic disease outcomes. From 1975 to 2016, over 90% of these epidemiological investigations on physical activity and mortality have used a single assessment of physical activity at baseline.^4^ Relating mortality risks to baseline physical activity levels does not account for within-person variation over the long term, potentially diluting the epidemiological associations. As physical activity behaviours are complex and vary over the life course,^5^ assessing within-person trajectories of physical activity over time would better characterise the association between physical activity and mortality.

Fewer studies have assessed physical activity trajectories over time and subsequent risks of mortality.^6^ ^7^ ^8^ ^9^ ^10^ ^11^ Some of these investigations have only included small samples of older adults, in either men or women. Importantly, most studies were limited by crude categorisations of physical activity patterns, without exposure calibration against objective measures with established validity. Many studies also do not adequately account for concurrent changes in other lifestyle risk factors—such as overall diet quality and body mass index—which might potentially confound the association between physical activity and mortality. This is important, as some studies have shown that associations between physical activity and weight gain are weak or inconsistent, suggesting that being overweight or obese might instead predict physical inactivity rather than the reverse.^12^ ^13^ Previous investigations have also not quantified the population impact of different physical activity trajectories over time on mortality. We examined associations of baseline and long term trajectories of within-person changes in physical activity on all cause, cardiovascular disease, and cancer mortality in a population based cohort study and quantified the number of preventable deaths from the observed physical activity trajectories.

## Methods

### Study population

The data for this investigation were from the European Prospective Investigation into Cancer and Nutrition-Norfolk (EPIC-Norfolk) study, comprising a baseline assessment and three follow-up assessments. The EPIC-Norfolk study is a population based cohort study of 25 639 men and women aged 40 to 79, resident in Norfolk, UK, and recruited between 1993 to 1997 from community general practices as previously described.^14^

After the baseline clinic assessment (1993 to 1997), the first follow-up (postal questionnaire) was conducted between 1995 and 1997 at a mean of 1.7 (SD 0.1) years after baseline, the second follow-up (clinic visit) took place 3.6 (0.7) years after baseline, and the third follow-up (postal questionnaire) was initiated 7.6 (0.9) years after the baseline clinic visit. All participants with repeated measures of physical activity (at least baseline and final follow-up assessments) were included, resulting in an analytical sample of 14 599 men and women.

### Assessment of physical activity

Habitual physical activity was assessed with a validated questionnaire, with a reference time frame of the past year.^15^ ^16^ The first question inquired about occupational physical activity, classified as five categories: unemployed, sedentary (eg, desk job), standing (eg, shop assistant, security guard), physical work (eg, plumber, nurse), and heavy manual work (eg, construction worker, bricklayer). The second open ended question asked about time spent (hours/week) on cycling, recreational activities, sports, or physical exercise, separately for winter and summer.

The validity of this instrument has previously been examined in an independent validation study, by using individually-calibrated combined movement and heart rate monitoring as the criterion method; physical activity energy expenditure (PAEE) increased through each of four ordinal categories of self reported physical activity comprising both occupational and leisure time physical activity.^15^ In this study, we disaggregated the index of total physical activity into its original two variables that were domain specific and conducted a calibration to PAEE using the validation dataset, in which the exact same instrument had been used (n=1747, omitting one study centre that had used a different instrument). Specifically, quasi-continuous and marginalised values of PAEE in units of kJ/kg/day were derived from three levels of occupational activity (unemployed or sedentary occupation; standing occupation; and physical or heavy manual occupation) and four levels of leisure time physical activity (none; 0.1 to 3.5 hours; 3.6 to 7 hours; and >7 hours per week). This regression procedure allows the domain specific levels of occupational and leisure time physical activity to have independent PAEE coefficients, while assigning a value of 0 kJ/kg/day to individuals with a sedentary (or no) occupation and reporting no leisure time physical activity (LTPA). The resulting calibration equation was: PAEE (kJ/kg/day) =0 (sedentary or no job) + 5.61 (standing job) + 7.63 (manual job) + 0 (no LTPA) + 3.59 (LTPA of 0.1 to 3.5 hours per week) + 7.17 (LTPA of 3.6 to 7 hours per week) + 11.26 (LTPA >7 hours per week).

### Assessment of covariates

Information about participants’ lifestyle and clinical risk factors were obtained at both clinic visits, carried out by trained nurses at baseline and 3.6 years later. Information collected during clinic visits included: age; height; weight; blood pressure; habitual diet; alcohol intake (units consumed per week); smoking status (never, former, and current smokers); physical activity; social class (unemployed, non-skilled workers, semiskilled workers, skilled workers, managers, and professionals); education level (none, General Certificate of Education (GCE) Ordinary Level, GCE Advanced Level, bachelor’s degree, and above); and medical history of heart disease, stroke, cancer, diabetes, fractures (wrist, vertebral, and hip), asthma, and other chronic respiratory conditions (bronchitis and emphysema). Additionally, updated information on heart disease, stroke, and cancer up to the final physical activity assessment (third follow-up) were also collected by using data from hospital episode statistics. This is a database containing details of all admissions, including emergency department attendances and outpatient appointments at National Health Service hospitals in England. Non-fasting blood samples were collected and refrigerated at 4°C until transported within a week of sampling to be assayed for serum triglycerides, total cholesterol, and high density lipoprotein cholesterol by using standard enzymatic techniques. We derived low density lipoprotein cholesterol by using the Friedewald equation.^17^

We assessed habitual dietary intake during the previous year by using validated 130 item food-frequency questionnaires administered at baseline and at the second clinic visit. The validity of this food-frequency questionnaire for major foods and nutrients was previously assessed against 16 day weighed diet records, 24 hour recall, and selected biomarkers in a subsample of this cohort.^18^ ^19^ We created a comprehensive diet quality score for each participant, separately for baseline and at follow-up, incorporating eight dietary components known to influence health and the risk of chronic disease.^20^ The composite diet quality score included: wholegrains, refined grains, sweetened confectionery and beverages, fish, red and processed meat, fruit and vegetables, sodium, and the ratio of unsaturated to saturated fatty acids from dietary intakes. We created tertiles for each dietary component and then scored these as −1, 0, or 1, with the directionality depending on whether the food or nutrient was associated with health risks or benefits.^20^ Scores from the eight dietary components were summed into an overall diet quality score which ranged from −8 to 8, with higher values representing a healthier dietary pattern. We also collected updated information on body weight and height from the two postal assessments (first and third follow-up).

### Mortality ascertainment

All participants were followed-up for mortality by the Office of National Statistics until the most recent censor date of 31 March 2016. Causes of death were confirmed by death certificates which were coded by nosologists according to ICD-9 (international classification of diseases, ninth revision) and ICD-10 (international classification of diseases, 10th revision). We defined cancer mortality and cardiovascular disease mortality by using codes ICD-9 140-208 or ICD-10 C00-C97 and ICD-9 400-438 or ICD-10 I10-I79, respectively.

### Statistical analysis

We used Cox proportional hazards regression models to derive hazard ratios and 95% confidence intervals. Individuals contributed person time from the date of the last physical activity assessment (third follow-up) until the date of death or censoring. We used all available assessments of physical activity to better represent long term habitual physical activity and used linear regression against elapsed time to derive an overall physical activity trajectory (ΔPAEE) for each individual. We used the resulting coefficient of the calibrated ΔPAEE values in kJ/kg/day/year, together with baseline PAEE, as mutually-adjusted exposure variables in the Cox regression models.

We created categories reflecting approximate tertiles of both baseline PAEE and ΔPAEE to investigate joint effects of baseline and long term trajectories of physical activity. We defined the categories of baseline PAEE as: low (PAEE=0 kJ/kg/day), medium (0<PAEE<8.4 kJ/kg/day), and high (PAEE≥8.4 kJ/kg/day). We defined the categories of ΔPAEE over time as: decreasers (ΔPAEE≤−0.20 kJ/kg/day/year), maintainers (−0.20<ΔPAEE<0.20 kJ/kg/day/year), and increasers (ΔPAEE≥0.20 kJ/kg/day/year). We then created joint exposure categories by cross-classifying the three baseline by the three trajectory categories, resulting in eight categories. The reference group was individuals with consistently low physical activity (by definition, there would be no exposure category comprising individuals declining from no baseline physical activity). We estimated the potential number of preventable deaths at the population level in each joint exposure category, using the absolute difference in adjusted mortality rates between the reference group (consistently inactive) and each joint exposure category, multiplied by the person years observed in the corresponding joint exposure category. We derived adjusted mortality rates by using multivariable exponential regression, with covariates used in the most comprehensively adjusted analytical model.

In model 1 we adjusted for: general demographics (age, sex, socioeconomic status, education level, and smoking status), dietary factors (total energy intake, overall diet quality, alcohol consumption), and medical history (asthma, chronic respiratory conditions, bone fractures, diabetes, heart disease, stroke, and cancer). Age, energy and alcohol intake, and diet quality were continuous variables. In model 2 we accounted for changes in the above covariates by further inclusion of updated variables at the second clinic visit (3.6 years later), as well as updated status of cardiovascular disease and cancer from hospital episode statistics up until the final physical activity assessment. In model 3 we further accounted for changes in body mass index by including continuous values of body mass index at baseline and at the final physical activity assessment. In model 4 we accounted for changes in blood pressure and lipids by further including continuous values of systolic and diastolic blood pressure, serum triglycerides, low density lipoprotein cholesterol, and high density lipoprotein cholesterol at baseline and at the second clinic visit.

We used height and weight measurements from the baseline and second clinic visit to calibrate self reported height and weight provided by the postal questionnaires. Self reported values were multiplied by the ratio of mean clinically-measured values and self-reported values. We imputed missing values of covariates at follow-up by using regression on their baseline values. A complete case analysis was conducted as a sensitivity analysis. Reverse causation owing to undiagnosed disease was mitigated by excluding participants who died within one year of the final physical activity assessment (beginning of follow-up for mortality) in all analyses. Predefined subgroups were age, sex, clinically-defined cut points of body mass index, and history of cardiovascular disease and cancer. We performed additional sensitivity analyses by excluding individuals with any period-prevalent chronic diseases (heart disease, stroke, and cancer) up to the final physical activity assessment, as well as excluding deaths occurring within two years of the final physical activity assessment. All analyses were performed by using Stata SE version 14.2.

### Patient and public involvement

Patients and members of the public were not formally involved in the design, analysis or interpretation of this study. Nonetheless, the research question in this article is of broad public health interest. The results of this study will be disseminated to study participants and the general public through the study websites, participant engagement events, seminars, and conferences.

## Results

### Study population

Among 14 599 participants with a mean baseline age of 58.0 (SD 8.8), followed for a median of 12.5 (interquartile range 11.9-13.2) years after the final physical activity assessment, there were 3148 deaths (950 from cardiovascular disease and 1091 from cancer) during 171 277 person years of follow-up. Table 1 shows the study population characteristics at the four assessment time points. On average, dietary factors such as total energy intake, alcohol consumption, and overall diet quality were similar at baseline and at the second clinic visit. The prevalence of diabetes, cardiovascular disease, cancer, and respiratory conditions increased over time. From baseline to the final follow-up assessment, mean body mass index increased from 26.1 kg/m^2^ to 26.7 kg/m^2^, and mean PAEE declined by 17% from 5.9 kJ/kg/day to 4.9 kJ/kg/day. The Pearson correlation coefficients were r=0.57 between PAEE at baseline and 1.7 years later; and r=0.45 between PAEE at baseline and 7.6 years later (final physical activity assessment).

**Table 1:** Study population characteristics at baseline and follow-up assessments. Values are means (SD) unless stated otherwise

Characteristic	Baseline		Follow-up		
			First	Second	Third
Period	1993-1997		1995-1999	1998-2000	2002-2004
Sample size (n)	14 599		11 889	11 408	14 599
Follow-up duration from baseline (years)	NA		1.7 (0.1)	3.6 (0.7)	7.6 (0.9)
Age (years)	58.0 (8.8)		60.1 (8.8)	62.0 (8.8)	65.5 (9.0)
Women (%)	56.6		56.6	56.6	56.6
Education level (%):					
Unemployed to semiskilled workers	15.1		NA	NA	NA
Skilled workers	38.1		NA	NA	NA
Managers and professionals	46.7		NA	NA	NA
Dietary factors:					
Energy intake (kcal/day)	2055 (593)		NA	1961 (554)	NA
Alcohol (units/week)*	7.1 (9.1)		NA	6.9 (9.0)	NA
Overall diet quality score†	0.2 (2.9)		NA	0.38 (2.5)	NA
Smoking status (%):					
Current	9.4		NA	6.3	NA
Former	41.0		NA	35.9	NA
Comorbidities (%):					
Diabetes	1.7		NA	3.2	NA
Heart disease	2.3		NA	3.0	5.3
Stroke	0.9		NA	2.2	3.4
Cancer	4.9		NA	7.6	9.6
Asthma	8.3		NA	10.5	NA
Chronic obstructive pulmonary disease	8.5		NA	10.7	NA
Bone fractures	6.6		NA	6.8	NA
Moderate to poor self rated health	15.8		NA	15.9	NA
Risk factors:					
Body mass index (kg/m^2^)	26.1 (3.8)		26.3 (3.8)	26.6 (3.9)	26.7 (4.2)
Systolic blood pressure (mm Hg)	134.1 (17.8)		NA	134.5 (17.9)	NA
Diastolic blood pressure (mm Hg)	82.0 (11.0)		NA	81.8 (11.1)	NA
Triglycerides (mmol/L)	1.76 (1.09)		NA	1.86 (1.07)	NA
Cholesterol (mmol/L):					
Total	6.14 (1.15)		NA	6.06 (1.15)	NA
HDL	1.43 (0.42)		NA	1.50 (0.46)	NA
LDL	3.94 (1.02)		NA	3.76 (1.04)	NA
PAEE (kJ/kg/day)	5.9 (4.7)		5.0 (4.6)	NA‡	4.9 (4.8)
ΔPAEE (kJ/kg/day/year)	NA		NA	NA	−0.11 (0.66)

NA=not available; HDL=high density lipoprotein; LDL=low density lipoprotein; PAEE=physical activity energy expenditure; ΔPAEE=trajectory of PAEE over time (annual rate of change), derived from within-individual regression of PAEE across all available physical activity assessments.

* 1unit=8 g

† range −8 to 8

‡ Physical activity at the second follow-up was not included in this analysis, since a different questionnaire was used.

### Associations of baseline and trajectories of physical activity with mortality

Table 2 shows that for each 1 kJ/kg/day/year increase in PAEE over time (ΔPAEE), the hazard ratios were: 0.78 (95% confidence interval 0.73 to 0.84) for all cause mortality, 0.75 (0.66 to 0.86) for cardiovascular disease mortality, and 0.88 (0.79 to 0.98) for cancer mortality (model 1). Progressive adjustments for time-updated covariates (model 2), changes in body mass index (model 3), and changes in blood pressure and blood lipids (model 4) did not attenuate the strength of the associations. In all models, baseline PAEE was also independently associated with lower mortality; for each 10 kJ/kg/day difference between individuals, hazard ratios were 0.70 (95% confidence interval 0.64 to 0.78) for all cause mortality, 0.69 (0.57 to 0.83) for cardiovascular disease mortality, and 0.83 (0.70 to 0.98) for cancer mortality (table 2, model 4). There was no evidence of an interaction between baseline PAEE and ΔPAEE for all mortality outcomes (P>0.6 from likelihood-ratio tests). The effect of PAEE averaged across all assessments on overall mortality, was 0.70 (0.62 to 0.78) for each 10 kJ/kg/day difference between individuals. For single time point exposure assessments, the inverse association of PAEE with mortality at the most recent assessment was stronger than that for baseline PAEE; hazard ratios were 0.68 (0.62 to 0.75) and 0.87 (0.80 to 0.94) for each 10 kJ/kg/day difference, respectively.

**Table 2:** Associations of mutually-adjusted baseline physical activity energy expenditure (PAEE) and trajectories of physical activity (ΔPAEE) with mortality. Values are hazard ratios (95% confidence intervals) unless stated otherwise

Outcome	Model			
	1*	2†	3‡	4§
Sample size (n)	14 599	14 599	14 587	13 360
Person years	171 277	171 277	171 138	156 075
**All cause mortality**				
Deaths	3148	3148	3145	2840
Baseline PAEE¶	0.70 (0.63 to 0.77)	0.71 (0.65 to 0.79)	0.72 (0.65 to 0.79)	0.70 (0.64 to 0.78)
ΔPAEE**	0.78 (0.73 to 0.84)	0.78 (0.73 to 0.84)	0.78 (0.73 to 0.84)	0.76 (0.71 to 0.82)
**Cardiovascular disease mortality**				
Deaths	950	950	949	850
Baseline PAEE	0.72 (0.60 to 0.86)	0.73 (0.61 to 0.88)	0.75 (0.62 to 0.89)	0.69 (0.57 to 0.83)
ΔPAEE	0.75 (0.66 to 0.86)	0.76 (0.66 to 0.86)	0.76 (0.67 to 0.87)	0.71 (0.62 to 0.82)
**Cancer mortality**				
Deaths	1091	1091	1090	977
Baseline PAEE	0.80 (0.69 to 0.94)	0.82 (0.70 to 0.96)	0.83 (0.70 to 0.97)	0.83 (0.70 to 0.98)
ΔPAEE	0.88 (0.79 to 0.98)	0.89 (0.79 to 0.99)	0.89 (0.79 to 0.99)	0.89 (0.79 to 1.00)

* Adjusted for age, sex, smoking status, education level, social class, self rated health, alcohol intake, energy intake, overall diet quality (comprising fruit and vegetables, red and processed meat, fish, wholegrains, refined grains, sweetened confectionery and beverages, ratio of unsaturated to saturated fat intake, and sodium) as well as for medical history at baseline (cardiovascular disease, cancer, diabetes, asthma, chronic obstructive pulmonary diseases, and bone fractures).

† Adjusted for covariates in model 1 and time-updated variables for smoking, alcohol intake, energy intake, diet quality and medical history at the second clinic visit, as well as period-prevalent heart disease, stroke and cancer from hospital episode statistics up to the final physical activity assessment (third follow-up).

‡ Adjusted for covariates in model 2 and body mass index at baseline and at the final physical activity assessment.

§ Adjusted for covariates in model 3 and systolic and diastolic blood pressure, triglycerides, low density lipoprotein cholesterol, and high density lipoprotein cholesterol at baseline and at the second clinic visit.

¶ For 10 kJ/kg/day differences in baseline PAEE.

** For 1 kJ/kg/day per year increase in ΔPAEE.

Sensitivity analyses excluding individuals with any period-prevalent heart disease, stroke, and cancer occurring up to the final physical activity assessment, as well as any deaths occurring within two years of this final assessment, showed similar associations with mortality for baseline PAEE and ΔPAEE, hazard ratios of 0.72 (95% confidence interval 0.63 to 0.81) for each 10 kJ/kg/day difference and 0.78 (0.71 to 0.86) for each 1 kJ/kg/day/year difference, respectively. Sensitivity analysis that used complete cases did not materially change the strength of associations (attenuation <5% for ΔPAEE estimates for all outcomes), but it attenuated the statistical significance for cancer mortality (supplementary table 1). Adjustments for occupational physical activity categories (sedentary, standing, physical, and heavy manual jobs) (supplementary table 2) slightly strengthened the association of ΔPAEE with cardiovascular disease mortality and attenuated the association with cancer mortality, whereas associations for baseline PAEE became stronger for both these outcomes.

### Stratified analyses

**Fig 1:**
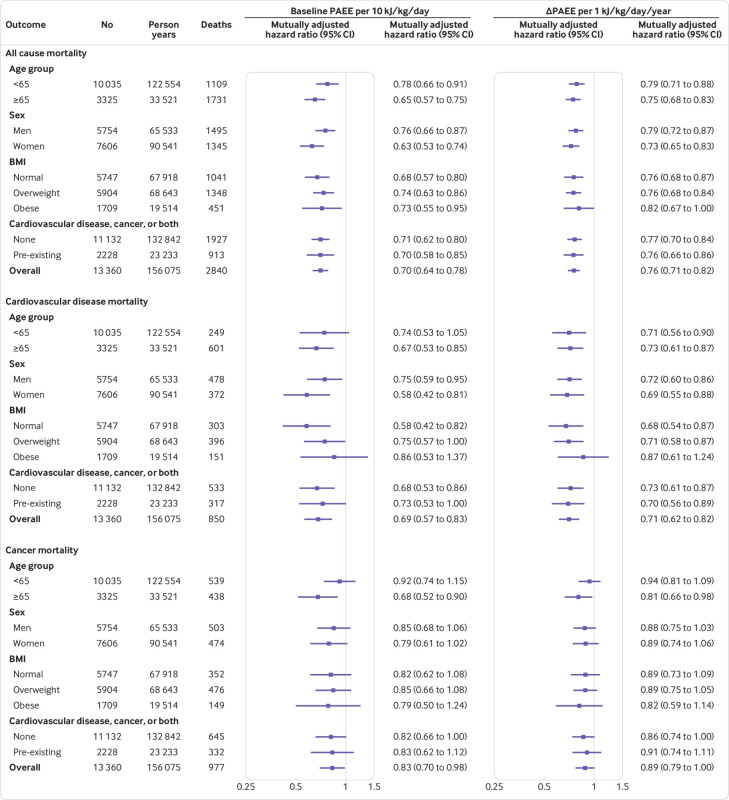
Associations of baseline and long term trajectories of physical activity energy expenditure (PAEE) with all cause, cardiovascular disease, and cancer mortality, stratified by age group, sex, body mass index (BMI), and disease status. Hazard ratios are mutually adjusted for both baseline PAEE and ΔPAEE, and are based on the most comprehensively adjusted model for changes in covariates, including medical history, diet quality, body mass index, blood pressure, and lipids (model 4 from table 2).

Figure 1 shows that, based on the most comprehensively-adjusted analytical model (model 4), significant inverse associations for baseline PAEE and ΔPAEE with all cause mortality persisted in all subgroups of age, sex, adiposity, and chronic disease status. Although tests for interaction were not statistically significant for any subgroup, the benefit of baseline PAEE on all cause mortality tended to be stronger in women (hazard ratio 0.63, 95% confidence interval 0.53 to 0.74) than men (0.76, 0.66 to 0.87; P=0.08). Baseline PAEE and ΔPAEE were not associated with cardiovascular disease mortality in individuals with obesity. In stratified analyses for cancer mortality, the longevity benefits of both baseline PAEE and ΔPAEE were only significant in older adults.

### Joint associations of baseline and trajectories of physical activity with mortality

**Fig 2:**
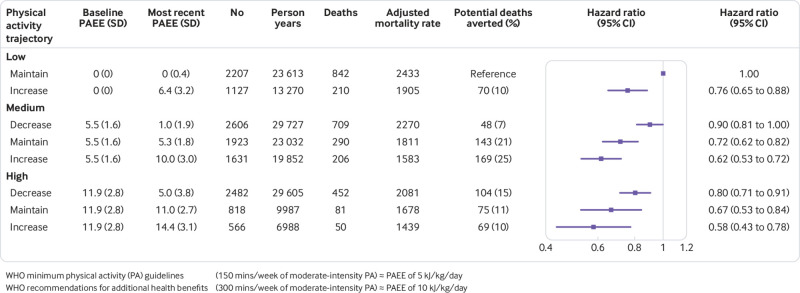
Joint associations of baseline and trajectories of physical activity energy expenditure (PAEE) with all cause mortality. Hazard ratios (HR) are based on the most comprehensively adjusted model for age, sex, sociodemographics, and changes in medical history, diet quality, body mass index, blood pressure, and lipids (model 4 from table 2). Adjusted mortality rates are expressed per 100 000 person years. WHO=World Health Organization.

Figure 2 shows that compared with individuals who were consistently inactive (low-maintainers), individuals with medium and high baseline physical activity who maintained these levels (medium-maintainers and high-maintainers) had significantly lower risks of all cause mortality, 28% and 33% respectively. Individuals with increasing physical activity trajectories experienced additional longevity benefits, including those with low baseline activity, as well as those with already high levels of baseline physical activity. Dose-response gradients were observed within and between strata of baseline physical activity levels. Within strata of low, medium, and high baseline physical activity, the risk of mortality was lower across ordinally increasing trajectories of: decreasers, maintainers, and increasers. Between strata of baseline physical activity, the risk of mortality decreased by 24% for low-increasers, 38% for medium-increasers, and 42% for high-increasers. Medium-decreasers and high-decreasers had 10% and 20% lower risks of mortality, respectively, compared with the reference group of low-maintainers.

### Estimation of population impact

Figure 2 shows that if the entire cohort remained inactive over time, an additional 24% of deaths (678 more than the observed 2840 deaths) would have potentially occurred. At the population level, the greatest number of potential deaths averted were in the medium-increasers and medium-maintainers, preventing 169 (25%) and 143 (21%) of the deaths associated with physical inactivity, respectively. All physical activity trajectories that culminated with meeting at least the minimum physical activity guidelines (equivalent to 5 kJ/kg/day) could potentially prevent 93% of the deaths associated with physical inactivity at the population level.

## Discussion

In this prospective cohort study with repeated assessments, we found protective associations for increasing physical activity trajectories against mortality from all causes, cardiovascular disease, and cancer, irrespective of past physical activity levels. These associations were also independent of levels and changes in several established risk factors such as overall diet quality, body mass index, medical history, blood pressure, triglycerides, and cholesterol. Both higher physical activity levels at baseline and increasing trajectories over time were protective against mortality. Notably, the strength of associations was similar in individuals with and without pre-existing cardiovascular disease and cancer. These results are encouraging, not least for middle aged and older adults with cardiovascular disease and cancer, who can still gain substantial longevity benefits by becoming more active, lending further support to the broad public health benefits of physical activity.

### Independent and joint effects of baseline and trajectories of physical activity

**Fig 3:**
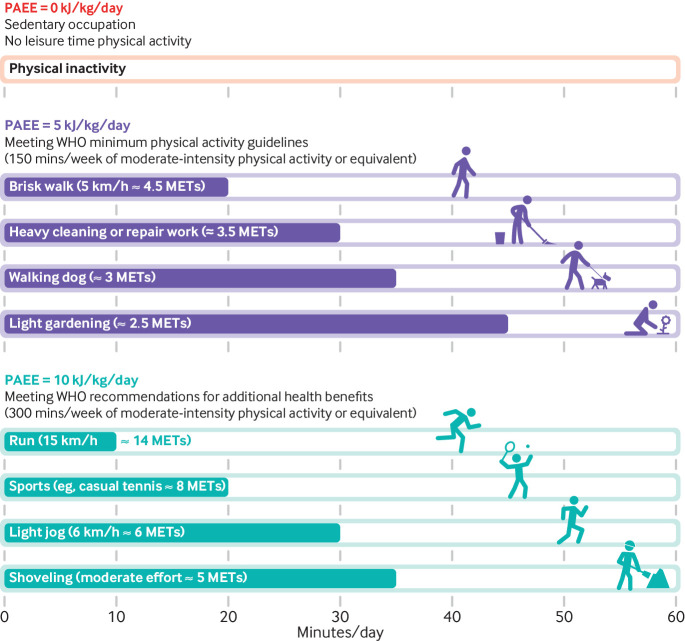
Physical activity energy expenditure (PAEE) of common activities performed during leisure time and at work. MET=metabolic equivalent of task. WHO=World Health Organization

The absence of an interaction between baseline physical activity levels and long term trajectories of physical activity on the risk of mortality suggests that the relative longevity benefit of increasing physical activity is consistent, irrespective of baseline levels. Increasing PAEE by 1 kJ/kg/day per year—equivalent to a trajectory of being inactive at baseline and then subsequently increasing physical activity to 5 and 10 kJ/kg/day, five and 10 years later, respectively—was associated with a 24% lower risk of all cause mortality. This gain in longevity from increasing physical activity over time, is in addition to the benefits already accrued from baseline physical activity, such as a 30% lower risk of mortality for a between-individual difference of 10 kJ/kg/day. For reference, 5 kJ/kg/day corresponds to the World Health Organization minimum physical activity guidelines of 150 minutes per week of moderate-intensity physical activity, and 10 kJ/kg/day corresponds to the WHO recommendations of 300 minutes per week of moderate-intensity physical activity for additional health benefits. Figure 3 shows how these levels of physical activity can be achieved in any number of ways during leisure time and at work, with the required duration depending on relative intensities of the activities undertaken.

The joint analyses of physical activity trajectories beginning from different baseline levels showed that adults who were already meeting at least the minimum physical activity recommendations (150 minutes per week of moderate physical activity), experience substantial longevity benefits by either maintaining or further increasing physical activity levels. This is evidenced by medium-maintainers and high-maintainers experiencing 28% and 33% lower risks of mortality, with an additional ~10% lower risk for increasers in both these baseline groups. 

Adults already meeting the equivalent of the higher WHO physical activity recommendations (300 minutes per week of moderate physical activity) still gain further longevity benefits by increasing physical activity levels to over 14 kJ/kg/day. This energy expenditure corresponds to approximately three times the recommended minimum (equivalent to 450 minutes per week of moderate physical activity). 

The joint analyses also revealed that some, but not all, of the longevity benefits from past physical activity levels are lost when previously active individuals decrease their activity levels. Compared with the consistently inactive, medium-decreasers and high-decreasers experienced 10% and 20% lower risks of mortality, respectively. However, these effects appear modest, compared with the 28% and 33% lower risks of mortality for medium-maintainers and high-maintainers, respectively. The low-increasers also experienced slightly lower risks of mortality than the high-decreasers (24% *v* 20% lower than the consistently-inactive reference group, respectively) but both had higher risks of mortality than the medium-maintainers (28% lower risk), despite all these three groups ending up at approximately the same physical activity level at the final exposure assessment. There might be several explanations for this, including: the relative importance of past versus more recent physical activity; differential factors that might have caused these specific physical activity trajectories in the first place, beyond differences in period-prevalent cardiovascular disease or cancer; and the degree to which physical activity levels were maintained or continued to change beyond the last exposure assessment until the date of final censoring.

### Population impact

Although mortality benefits were greatest in the high-increasers (hazard ratio of 0.58), the fraction of potential deaths averted at the population level were greatest for the medium-increasers (25%) and the medium-maintainers (21%), in contrast with 10% for the high-increasers. This is owing to the combination of a moderately strong aetiological association (hazard ratio of 0.72) and a greater prevalence of medium-maintainers (23 032 person years, 15% of total person years), compared with high-increasers (6988 person years, 4% of total). All physical activity trajectories during middle to late adulthood that culminate with meeting at least the minimum physical activity guidelines could potentially prevent 93% of deaths attributable to physical inactivity. The last 7% were the 48 deaths potentially prevented by the medium-decreasers, who as a group did not meet the minimum physical activity guidelines at the final exposure assessment. Had this group maintained their baseline physical activity levels, an additional 136 deaths (nearly three times as many) may have been prevented. Comparatively fewer, yet still an extra 119 deaths (twice as many) could have been prevented if high-decreasers had maintained their baseline physical activity levels. These two groups with declining physical activity levels were also the most prevalent trajectories in the cohort. Thus, in addition to shifting the population towards meeting the minimum physical activity recommendations, public health efforts should also focus on the maintenance of physical activity levels, specifically preventing declines over middle and late life. The WHO minimum guidelines of 150 minutes per week of moderate-intensity physical activity appears to be a realistic public health target, given that these levels were observed to be broadly attainable at the population level. Individuals with existing chronic conditions such as cardiovascular disease and cancer—for whom our study has also shown to gain longevity benefits—might choose to engage in commensurably lower-intensity activities but for a longer duration (fig 3). Further research is, however, needed to specifically ascertain the health benefits of lower-intensity physical activity in both healthy individuals and those with major chronic diseases.^21^

### Comparisons with existing studies

This study also showed that the longevity benefits of increasing physical activity are independent of intermediary changes in several established risk factors, including body mass index, blood pressure, triglycerides, and cholesterol. These results are interesting, relative to other studies that have shown considerable attenuation of the strength of associations after adjustment for similar cardiometabolic biomarkers.^2^ ^22^ In our study, it is somewhat surprising that the inverse associations of physical activity with cardiovascular disease mortality were not attenuated (but rather strengthened) after adjusting for established cardiometabolic risk factors. These findings support research into other potential mechanisms, including vascular function,^23^ novel lipids,^24^ and autonomic nervous system activity,^25^ through which physical activity might protect against cardiovascular disease. In our study, the protective associations of physical activity were stronger for cardiovascular disease mortality than for cancer mortality, suggesting that longevity benefits were primarily driven through the prevention of cardiovascular-related deaths. Adjustment for occupational physical activity strengthened the inverse associations with cardiovascular disease mortality for both baseline PAEE and ΔPAEE; this was also the case for the association between baseline PAEE and cancer mortality, but the association between ΔPAEE and cancer mortality was attenuated. The existing body of evidence, from a meta-analysis of nine international cohort studies also reported stronger inverse associations for cardiovascular disease mortality, compared with cancer mortality.^26^ ^27^ The weaker associations with cancer mortality might reflect the notion that cancers are a collection of neoplastic diseases, which might be aetiologically diverse and characterised by separate pathophysiologies.^1^

Our results for the association of baseline physical activity with mortality were broadly similar to those reported in the literature, although our estimates have accounted for changes in physical activity over time, which to some degree would correct for regression dilution bias.^28^ In a pooled analysis of data from high income countries within the Prospective Urban Rural Epidemiologic (PURE) study,^29^ medium baseline physical activity (150 to 750 minutes per week of moderate-intensity physical activity) was associated with a 31% lower risk of mortality. This is similar to our estimates of a 30% lower risk of mortality for a 10 kJ/kg/day difference between individuals (equivalent to 300 minutes per week of moderate-intensity physical activity). 

Another pooled analysis examining the dose-response relation between baseline leisure time physical activity and mortality also reported lower mortality risks of between 31% to 37% at a comparable volume of physical activity.^30^ Comparisons with previous studies, examining specifically the changes and patterns of physical activity over time on mortality, are difficult owing to methodological and analytical heterogeneity between studies, precluding the synthesis of published results using meta-analytic methods. There was considerable variation in the operational definitions of the “changes” in physical activity over time. Some classified “changes” as increases and decreases, compared with unchanged physical activity irrespective of baseline levels^31^; others grouped varying activity levels over time as “mixed patterns,”^4^ potentially obscuring the benefits for individuals who improved physical activity levels over time; yet others used a reference group of the “consistently-active.”^6^ ^9^ Furthermore, the time periods for studying these physical activity trajectories were also variable, with some studies examining short term changes within one to two years,^32^ ^33^ and others examining changes over 10 years.^6^ ^10^ ^11^ Nonetheless, the relative risks of our high-maintainer and medium-maintainer groups (with hazard ratios of 0.67 and 0.72, respectively) were broadly in the ranges of the consistently-active groups reported in previous studies.^7^ ^8^ ^11^ Future work examining physical activity trajectories over time on health outcomes could consider pooling of individual-level harmonised data from compatible studies with repeated follow-up assessments, ideally combined with external calibration; this would enable standardisation of exposure definitions and analytical approaches.

### Strengths and limitations of the study

On balance, we present a comprehensive analysis, examining longitudinal physical activity trajectories in a large cohort with long follow-up for mortality, and quantified the population health impact from different physical activity trajectories. To overcome limitations in the majority of studies which have predominantly examined mortality associations with physical activity assessed at a single time point, we incorporated repeated measures of physical activity calibrated against objective measurements of individually-calibrated combined movement and heart rate monitoring. The use of longitudinal, within-individual trajectories of physical activity over time also precludes any confounding by time-invariant factors such as genetics. Our approach offers a stronger operationalisation of physical activity exposures, representing a method which can be used in future longitudinal studies investigating the associations between physical activity and subsequent health outcomes. Our study showed robust protective associations between physical activity and mortality, even after controlling for established risk factors, such as overall diet quality, body mass index, blood pressure, triglycerides, and cholesterol. 

Some limitations of our study are that the analytical sample comprised of individuals who were available for follow-up approximately a decade after initial recruitment. Thus, a healthy cohort effect cannot be excluded. This, however, would only serve to render our findings more conservative. As the study was observational, residual confounding owing to unmeasured factors might still be possible. However, it would be virtually impossible to study the effects of habitual physical activity on mortality in a randomised controlled trial, and the observational nature of this study broadly shows the attainable longevity benefits of physical activity trajectories observed in the real world.

### Conclusion

We showed that middle aged and older adults, including those with cardiovascular disease and cancer, stand to gain substantial longevity benefits by becoming more physically active, irrespective of past physical activity levels and established risk factors—including overall diet quality, body mass index, blood pressure, triglycerides, and cholesterol. Maintaining or increasing physical activity levels from a baseline equivalent to meeting the minimum public health recommendations has the greatest population health impact, with these trajectories being responsible for preventing nearly one in two deaths associated with physical inactivity. In addition to shifting the population towards meeting the minimum physical activity recommendations, public health efforts should also focus on the maintenance of physical activity levels, specifically preventing declines over mid to late life.

**What is already known on this topic**

Physical activity assessed at a single time point is associated with lower risks of mortality from all causes, cardiovascular disease, and cancer
Fewer studies have examined long term changes in physical activity and quantified the population health impact of different activity trajectories

**What this study adds**

Middle aged and older adults, including those with cardiovascular disease and cancer, stand to gain substantial longevity benefits by becoming more physically active, regardless of past activity levels, and changes in established risk factors, including overall diet quality, bodyweight, blood pressure, triglycerides, and cholesterol
At the population level, meeting and maintaining at least the minimum public health recommendations (150 minutes per week of moderate-intensity physical activity) would potentially prevent 46% of deaths associated with physical inactivity
Public health strategies should shift the population towards meeting the minimum recommendations, and importantly, focus on preventing declines in physical activity during middle and late life

## Web Extra

Extra material supplied by the author

## Notes

Contributors: KTK, RL, and NW contributed to the design of the EPIC-Norfolk study. AM and SB conceptualised the design of the present analysis and analysed the data. AM wrote the first draft of the manuscript. All authors had full access to the data in the study and can take responsibility for the integrity of the data and the accuracy of the data analysis. AM and SB and are the guarantors. The corresponding author attests that all listed authors meet authorship criteria and that no others meeting the criteria have been omitted.

Ethical approval: The study was approved by the Norfolk District Health Authority Ethics Committee and adhered to the World Medical Association’s Declaration of Helsinki.

Patient consent: All participants gave written informed consent before enrolment in the study.

Data sharing: No additional data are available.

The lead author (AM) affirms that this manuscript is an honest, accurate, and transparent account of the study being reported; that no important aspects of the study have been omitted; and that any discrepancies from the study as planned have been explained.

## References

[1] Moore SC Lee I-M Weiderpass E Association of Leisure-Time Physical Activity With Risk of 26 Types of Cancer in 1.44 Million Adults. JAMA Intern Med 2016;176:816-25. 10.1001/jamainternmed.2016.1548. 27183032 PMC5812009

[2] Mora S Cook N Buring JE Ridker PM Lee I-M Physical activity and reduced risk of cardiovascular events: potential mediating mechanisms. Circulation 2007;116:2110-8. 10.1161/CIRCULATIONAHA.107.729939. 17967770 PMC2117381

[3] Lee I-M Shiroma EJ Lobelo F Puska P Blair SN Katzmarzyk PT Lancet Physical Activity Series Working Group Effect of physical inactivity on major non--communicable diseases worldwide: an analysis of burden of disease and life expectancy. Lancet 2012;380:219-29. 10.1016/S0140-6736(12)61031-9. 22818936 PMC3645500

[4] Bauman AE Grunseit AC Rangul V Heitmann BL Physical activity, obesity and mortality: does pattern of physical activity have stronger epidemiological associations? BMC Public Health 2017;17:788. 10.1186/s12889-017-4806-6. 28982371 PMC5629749

[5] Barengo NC Nissinen A Tuomilehto J Pekkarinen H Twenty-five-year trends in physical activity of 30- to 59-year-old populations in eastern Finland. Med Sci Sports Exerc 2002;34:1302-7. 10.1097/00005768-200208000-00011. 12165685

[6] Byberg L Melhus H Gedeborg R Total mortality after changes in leisure time physical activity in 50 year old men: 35 year follow-up of population based cohort. BMJ 2009;338:b688. 10.1136/bmj.b688. 19264819 PMC2654773

[7] Gregg EW Cauley JA Stone K Study of Osteoporotic Fractures Research Group Relationship of changes in physical activity and mortality among older women. JAMA 2003;289:2379-86. 10.1001/jama.289.18.2379. 12746361

[8] Schnohr P Scharling H Jensen JS Changes in leisure-time physical activity and risk of death: an observational study of 7,000 men and women. Am J Epidemiol 2003;158:639-44. 10.1093/aje/kwg207. 14507599

[9] Bijnen FC Feskens EJ Caspersen CJ Nagelkerke N Mosterd WL Kromhout D Baseline and previous physical activity in relation to mortality in elderly men: the Zutphen Elderly Study. Am J Epidemiol 1999;150:1289-96. 10.1093/oxfordjournals.aje.a009960. 10604771

[10] Wannamethee SG Shaper AG Walker M Changes in physical activity, mortality, and incidence of coronary heart disease in older men. Lancet 1998;351:1603-8. 10.1016/S0140-6736(97)12355-8. 9620713

[11] Paffenbarger RS Jr Hyde RT Wing AL Lee IM Jung DL Kampert JB The association of changes in physical-activity level and other lifestyle characteristics with mortality among men. N Engl J Med 1993;328:538-45. 10.1056/NEJM199302253280804. 8426621

[12] Pedisic Z Grunseit A Ding D High sitting time or obesity: Which came first? Bidirectional association in a longitudinal study of 31,787 Australian adults. Obesity (Silver Spring) 2014;22:2126-30. 10.1002/oby.20817. 24943057 PMC4265269

[13] Golubic R Ekelund U Wijndaele K Rate of weight gain predicts change in physical activity levels: a longitudinal analysis of the EPIC-Norfolk cohort. Int J Obes (Lond) 2013;37:404-9. 10.1038/ijo.2012.58. 22531093 PMC3635037

[14] Day N Oakes S Luben R EPIC-Norfolk: study design and characteristics of the cohort. European Prospective Investigation of Cancer. Br J Cancer 1999;80(Suppl 1):95-103. 10466767

[15] Peters T Brage S Westgate K InterAct Consortium Validity of a short questionnaire to assess physical activity in 10 European countries. Eur J Epidemiol 2012;27:15-25. 10.1007/s10654-011-9625-y. 22089423 PMC3292724

[16] Khaw K-T Jakes R Bingham S Work and leisure time physical activity assessed using a simple, pragmatic, validated questionnaire and incident cardiovascular disease and all-cause mortality in men and women: The European Prospective Investigation into Cancer in Norfolk prospective population study. Int J Epidemiol 2006;35:1034-43. 10.1093/ije/dyl079. 16709620

[17] Friedewald WT Levy RI Fredrickson DS Estimation of the concentration of low-density lipoprotein cholesterol in plasma, without use of the preparative ultracentrifuge. Clin Chem 1972;18:499-502. 4337382

[18] Bingham SA Gill C Welch A Validation of dietary assessment methods in the UK arm of EPIC using weighed records, and 24-hour urinary nitrogen and potassium and serum vitamin C and carotenoids as biomarkers. Int J Epidemiol 1997;26(Suppl 1):S137-51. 10.1093/ije/26.suppl_1.S137. 9126542

[19] Bingham SA Welch AA McTaggart A Nutritional methods in the European Prospective Investigation of Cancer in Norfolk. Public Health Nutr 2001;4:847-58. 10.1079/PHN2000102. 11415493

[20] Micha R Peñalvo JL Cudhea F Imamura F Rehm CD Mozaffarian D Association Between Dietary Factors and Mortality From Heart Disease, Stroke, and Type 2 Diabetes in the United States. JAMA 2017;317:912-24. 10.1001/jama.2017.0947. 28267855 PMC5852674

[21] Chastin SFM De Craemer M De Cocker K How does light-intensity physical activity associate with adult cardiometabolic health and mortality? Systematic review with meta-analysis of experimental and observational studies. Br J Sports Med 2019;53:370-6. 10.1136/bjsports-2017-097563 29695511 PMC6579499

[22] Sattelmair J Pertman J Ding EL Kohl HW 3rd Haskell W Lee IM Dose response between physical activity and risk of coronary heart disease: a meta-analysis. Circulation 2011;124:789-95. 10.1161/CIRCULATIONAHA.110.010710. 21810663 PMC3158733

[23] Hawkins M Gabriel KP Cooper J Storti KL Sutton-Tyrrell K Kriska A The impact of change in physical activity on change in arterial stiffness in overweight or obese sedentary young adults. Vasc Med 2014;19:257-63. 10.1177/1358863X14536630. 24879662 PMC4877277

[24] Li S Guo Y-L Zhao X Novel and traditional lipid-related biomarkers and their combinations in predicting coronary severity. Sci Rep 2017;7:360. 10.1038/s41598-017-00499-9. 28336922 PMC5428477

[25] Barthélémy J-C Pichot V Dauphinot V Autonomic nervous system activity and decline as prognostic indicators of cardiovascular and cerebrovascular events: the ‘PROOF’ Study. Study design and population sample. Associations with sleep-related breathing disorders: the ‘SYNAPSE’ Study. Neuroepidemiology 2007;29:18-28. 10.1159/000108914. 17898520

[26] Hupin D Roche F Gremeaux V Even a low-dose of moderate-to-vigorous physical activity reduces mortality by 22% in adults aged ≥60 years: a systematic review and meta-analysis. Br J Sports Med 2015;49:1262-7. 10.1136/bjsports-2014-094306. 26238869

[27] Hupin D Edouard P Gremeaux V Physical activity to reduce mortality risk. Eur Heart J 2017;38:1534-7. 10.1093/eurheartj/ehx236. 29048470

[28] Clarke R Shipley M Lewington S Underestimation of risk associations due to regression dilution in long-term follow-up of prospective studies. Am J Epidemiol 1999;150:341-53. 10.1093/oxfordjournals.aje.a010013. 10453810

[29] Lear SA Hu W Rangarajan S The effect of physical activity on mortality and cardiovascular disease in 130 000 people from 17 high-income, middle-income, and low-income countries: the PURE study. Lancet 2017;390:2643-54. 10.1016/S0140-6736(17)31634-3. 28943267

[30] Arem H Moore SC Patel A Leisure time physical activity and mortality: a detailed pooled analysis of the dose-response relationship. JAMA Intern Med 2015;175:959-67. 10.1001/jamainternmed.2015.0533. 25844730 PMC4451435

[31] Hulsegge G Looman M Smit HA Daviglus ML van der Schouw YT Verschuren WM Lifestyle changes in young adulthood and middle age and risk of cardiovascular disease and all-cause mortality: The doetinchem cohort study. J Am Heart Assoc 2016;5:1-11. 10.1161/JAHA.115.002432. 26764411 PMC4859361

[32] Talbot LA Morrell CH Fleg JL Metter EJ Changes in leisure time physical activity and risk of all-cause mortality in men and women: the Baltimore Longitudinal Study of Aging. Prev Med 2007;45:169-76. 10.1016/j.ypmed.2007.05.014. 17631385

[33] Yates T Haffner SM Schulte PJ Association between change in daily ambulatory activity and cardiovascular events in people with impaired glucose tolerance (NAVIGATOR trial): a cohort analysis. Lancet 2014;383:1059-66. 10.1016/S0140-6736(13)62061-9. 24361242

