# Supplementary material for: Physical activity trajectories and mortality: population based cohort study
Source: BMJ. 2019 Jun 26;365:l2323. doi: 10.1136/bmj.l2323 (PMC6592407; doi:10.1136/bmj.l2323)
Supplement: Supplementary file 1 — Supplementary materials: Supplementary tables 1 and 2 [file moka047584.ww1.pdf]

Supplementary Table 1. Analysis of complete cases for associations of mutually-adjusted baseline physical activity energy expenditure (PAEE) and trajectories of physical activity (ΔPAEE) with mortality

	**Exposures**<br>**Baseline PAEE** (per 10 *kJ/kg/day*)<br>**Δ PAEE** (per 1 *kJ/kg/day/year*)	**Model 1:**<br>Adjustment for **baseline covariates & diet**<br>n=14,599<br>171,277 person-years	**Model 2:**<br>Additional adjustments **for changes in covariates & diet**<br>n= 9,222<br>108,751 person-years	**Model 3:**<br>Additional adjustments **for changes in body mass index**<br>n= 9,215<br>108,668 person-years	**Model 4:**<br>Additional adjustments **for changes in blood pressure and lipids**<br>n= 8,163<br>96,342 person-years
**All-cause Mortality**		3,148 deaths	1,923 deaths	1,921 deaths	1,686 deaths
	Baseline PAEE	0.70 (0.63 to 0.77)***	0.75 (0.67 to 0.85)***	0.76 (0.67 to 0.85)***	0.74 (0.65 to 0.85)***
	Δ PAEE	0.78 (0.73 to 0.84)***	0.81 (0.74 to 0.88)***	0.81 (0.74 to 0.88)***	0.79 (0.72 to 0.87)***
**CVD Mortality**		950 deaths	569 deaths	569 deaths	497 deaths
	Baseline PAEE	0.72 (0.60 to 0.86)***	0.77 (0.61 to 0.96)*	0.77 (0.62 to 0.97)*	0.70 (0.55 to 0.90)**
	Δ PAEE	0.75 (0.66 to 0.86)***	0.79 (0.67 to 0.93)**	0.79 (0.67 to 0.93)**	0.76 (0.63 to 0.90)**
**Cancer Mortality**		1,091 deaths	693 deaths	692 deaths	601 deaths
	Baseline PAEE	0.80 (0.69 to 0.94)**	0.87 (0.72 to 1.06)	0.87 (0.72 to 1.06)	0.91 (0.74 to 1.12)
	Δ PAEE	0.88 (0.79 to 0.98)*	0.93 (0.81 to 1.07)	0.92 (0.80 to 1.06)	0.93 (0.80 to 1.08)

All hazard ratios (HRs) and 95% confidence intervals (CIs) are for 10 kJ/kg/day differences in baseline PAEE; and for 1 kJ/kg/day per year increase in ΔPAEE.
ΔPAEE = Trajectory of physical activity energy expenditure (PAEE) over time (annual rate of change), derived from within-individual regression of PAEE across all available physical activity assessments. CVD = cardiovascular disease.
Model 1 is adjusted for age, sex, smoking status, education level, social class, self-rated health, alcohol intake, energy intake, overall diet quality (comprising fruit & vegetables, red & processed meat, fish, wholegrains, refined grains, sugar-sweetened snacks and beverages, ratio of unsaturated-to-saturated fat intake, and sodium) as well as for medical history at baseline (cardiovascular disease, cancer, diabetes, asthma, chronic obstructive pulmonary diseases, and bone fractures).
Model 2 is adjusted for covariates in Model 1 + time-updated variables for smoking, alcohol intake, energy intake, diet quality and medical history at the 2nd clinic visit, as well as period-prevalent heart disease, stroke and cancer from hospital episode statistics up to the final physical activity assessment (3rd follow-up).
Model 3 is adjusted for covariates in Model 2 + body mass index at baseline and at the final physical activity assessment
Model 4 is adjusted for covariates in Model 3 + systolic and diastolic blood pressure, serum triglycerides, LDL-cholesterol, and HDL-cholesterol at baseline and at the 2nd clinic visit.
There was no evidence of an interaction between baseline PAEE and ΔPAEE: p=0.50, p=0.68, and p=0.27 for all-cause, cardiovascular and cancer mortality, respectively (based on likelihood ratio tests using Model 4). Asterisks indicate level of statistical significance: *p<0.05; **p≤0.01; ***p≤0.001.

Supplementary Table 2. Associations of mutually-adjusted baseline physical activity energy expenditure (PAEE) and trajectories of physical activity (ΔPAEE) with mortality, with further adjustments for occupational physical activity

	**Exposures**<br>**Baseline PAEE** (per 10 *kJ/kg/day*)<br>**Δ PAEE** (per 1 *kJ/kg/day*)	**Model 1:**<br>Adjustment for **baseline covariates & diet**<br>n=14,599<br>171,277 person-years	**Model 2:**<br>Additional adjustments **for changes in covariates & diet**<br>n= 14,599<br>171,277 person-years	**Model 3:**<br>Additional adjustments **for changes in body mass index**<br>n= 14,587<br>171,138 person-years	**Model 4:**<br>Additional adjustments **for changes in blood pressure and lipids**<br>n= 13,360<br>156,075 person-years
**All-cause Mortality**		3,148 deaths	3,148 deaths	3,145 deaths	2,840 deaths
	Baseline PAEE	0.71 (0.62 to 0.81)***	0.72 (0.63 to 0.82)***	0.73 (0.64 to 0.83)***	0.73 (0.64 to 0.84)***
	Δ PAEE	0.81 (0.73 to 0.89)***	0.80 (0.73 to 0.89)***	0.81 (0.73 to 0.89)***	0.80 (0.72 to 0.88)***
**CVD Mortality**		950 deaths	950 deaths	949 deaths	850 deaths
	Baseline PAEE	0.66 (0.52 to 0.85)***	0.67 (0.52 to 0.86)**	0.69 (0.54 to 0.88)**	0.68 (0.52 to 0.89)**
	Δ PAEE	0.69 (0.57 to 0.82)***	0.68 (0.57 to 0.82)***	0.69 (0.57 to 0.83)***	0.66 (0.54 to 0.80)***
**Cancer Mortality**		1,091 deaths	1,091 deaths	1,090 deaths	977 deaths
	Baseline PAEE	0.79 (0.63 to 0.98)*	0.79 (0.64 to 0.99)*	0.80 (0.64 to 1.00)	0.78 (0.62 to 0.99)*
	Δ PAEE	0.95 (0.82 to 1.11)	0.95 (0.81 to 1.10)	0.95 (0.82 to 1.10)	0.94 (0.80 to 1.10)

All hazard ratios (HRs) and 95% confidence intervals (CIs) are for 10 kJ/kg/day differences in baseline PAEE; and for 1 kJ/kg/day per year increase in ΔPAEE.
ΔPAEE = Trajectory of physical activity energy expenditure (PAEE) over time (annual rate of change), derived from within-individual regression of PAEE across all available physical activity assessments. CVD = cardiovascular disease.
Model 1 is adjusted for age, sex, smoking status, education level, social class, self-rated health, alcohol intake, energy intake, overall diet quality (comprising fruit & vegetables, red & processed meat, fish, wholegrains, refined grains, sugar-sweetened snacks and beverages, ratio of unsaturated-to-saturated fat intake, and sodium) as well as for medical history at baseline (cardiovascular disease, cancer, diabetes, asthma, chronic obstructive pulmonary diseases, and bone fractures).
Model 2 is adjusted for covariates in Model 1 + time-updated variables for smoking, alcohol intake, energy intake, diet quality and medical history at the 2nd clinic visit, as well as period-prevalent heart disease, stroke and cancer from hospital episode statistics up to the final physical activity assessment (3rd follow-up).
Model 3 is adjusted for covariates in Model 2 + body mass index at baseline and at the final physical activity assessment
Model 4 is adjusted for covariates in Model 3 + systolic and diastolic blood pressure, serum triglycerides, LDL-cholesterol, and HDL-cholesterol at baseline and at the 2nd clinic visit. All models are further adjusted for occupational physical activity categories (sedentary or no occupation; standing work; physical work; and heavy manual labour) at baseline and at the final physical activity assessment. There was no evidence of an interaction between baseline PAEE and ΔPAEE: p=0.83, p=0.83, and p=0.90 for all-cause, cardiovascular and cancer mortality, respectively (based on likelihood ratio tests on Model 4). Asterisks indicate level of statistical significance: *$p<0.05$; **$p\le0.01$; ***$p\le0.001$
